# Impacts of travel activity and urbanicity on exposures to ambient oxides of nitrogen and on exposure disparities

**DOI:** 10.1007/s11869-014-0275-6

**Published:** 2014-07-10

**Authors:** Sashikanth Gurram, Amy Lynette Stuart, Abdul Rawoof Pinjari

**Affiliations:** 1 Department of Environmental and Occupational Health, University of South Florida, 13201 Bruce B. Downs Blvd., MDC 56, Tampa, FL 33612 USA; 2 Department of Civil and Environmental Engineering, University of South Florida, Tampa, USA; 3 School of Population Health, University of Western Australia, Crawley, Australia

**Keywords:** Traffic pollution, Human activity patterns, Environmental inequality, Urban form, Exposure error

## Abstract

Daily exposures to ambient oxides of nitrogen were estimated here for residents of Hillsborough County, FL. The 2009 National Household Travel Survey provided geocoded data on fixed activity locations during each person-day sampled. Routes between activity locations were calculated from transportation network data, assuming the quickest travel path. To estimate daily exposure concentrations for each person-day, the exposure locations were matched with diurnally and spatially varying ambient pollutant concentrations derived from CALPUFF dispersion model results. The social distribution of exposures was analyzed by comparing frequency distributions of grouped daily exposure concentrations and by regression modeling. To investigate exposure error, the activity-based exposure estimates were also compared with estimates derived using residence location alone. The mean daily activity-based exposure concentration for the study sample was 17 μg/m^3^, with values for individual person-day records ranging from 7.0 to 43 μg/m^3^. The highest mean exposure concentrations were found for the following groups: black (20 μg/m^3^), below poverty (18 μg/m^3^), and urban residence location (22 μg/m^3^). Urban versus rural residence was associated with the largest increase in exposure concentration in the regression (8.3 μg/m^3^). Time in nonresidential activities, including travel, was associated with an increase of 0.2 μg/m^3^ per hour. Time spent travelling and at nonresidential locations contributed an average of 6 and 24 %, respectively, to the daily estimate. A mean error of 3.6 %, with range from −64 to 58 %, was found to result from using residence location alone. Exposure error was highest for those who travel most, but lowest for the sociodemographic subgroups with higher mean exposure concentrations (including blacks and those from below poverty households). This work indicates the importance of urbanicity to social disparities in activity-based air pollution exposures. It also suggests that exposure error due to using residence location may be smaller for more exposed groups.

## Introduction

Estimation of human exposures to air pollution is important to researchers and practitioners in the fields of air quality management, environmental epidemiology, and urban design. Exposure estimation requires characterization of pollutant concentrations when and where a person or group spends time (Ott 1982). Although personal monitoring has long been used to determine exposures in the field of air pollution epidemiology (Dockery and Spengler 1981), it is time and cost intensive, resulting in small sample sizes that may be limited for representing a general population (Jerrett et al. 2005; Pekkanen and Pearce 2001). Hence, methods of estimating exposures for a large group of people are needed for population-level risk assessment and policy decisions.

For large-sample studies, exposures to air pollutants have often been estimated using residence address to represent the location of exposure. Concentrations measured at fixed monitoring sites or concentration surrogates (such as nearby traffic counts) are used to derive exposures at the residence locations (Huang and Batterman 2000; Meng et al. 2007; von Klot et al. 2009). Although this is a relatively simple and generalizable approach that can be applied in the context of available data, it is recognized that human activity patterns may be particularly important for explaining exposure variation (Klepeis et al. 2001; National Center for Environmental Assessment et al. 2011; Ott et al. 1986). Hence, exposure error and misclassification are concerns, with potential outcomes of inaccurate health and environmental impact assessments and policy interventions (Huang and Batterman 2000; Krzyzanowski 1997; Özkaynak et al. 1986; Sheppard et al. 2012; Thomas et al. 1993; Zeger et al. 2000).

As a result, studies have investigated the use of more refined estimates of population location and concentration to represent personal exposures, through methods that characterize or apply patterns of human activity (e.g., from time activity dairies) and microenvironment concentrations (Dons et al. 2011; Kornartit et al. 2010; Lai et al. 2004). These methods often improve the estimate of group-level and personal exposures but remain substantially limited in the characterization of spatiotemporal variations in concentrations and activities. A few recent case studies have begun using detailed activity-travel patterns derived from travel surveys or activity-based models, coupled with air pollution modeling, to estimate air quality exposures or health impacts (Dons et al. 2014; Gariazzo et al. 2011; Hankey et al. 2012; Hatzopoulou and Miller 2010), including analysis of exposure error (Dhondt et al. 2012; Setton et al. 2011), exposure inequality (Marshall et al. 2006; Marshall 2008), impacts of travel (Beckx et al. 2009; de Nazelle et al. 2013; Zhang and Batterman 2013), urban form (Stone et al. 2007), and transportation policies (Dhondt et al. 2013). Nonetheless, the literature remains sparse, and additional case studies applying and improving these methods are needed. Additionally, limited literature exists on the social distribution of exposure error.

This study is part of an ongoing project that aims to enhance the current understanding on exposures to traffic-related air pollution, specifically on the social distribution of exposure and impacts of urban design (Evans and Stuart 2011; Fridh and Stuart 2014; Stuart et al. 2009; Stuart and Zeager 2011; Yu and Stuart 2013). Here, we investigate impacts of activities and urban design factors on exposures and exposure disparities. We also estimate the error introduced by use of residence location only versus detailed spatiotemporal activity on exposure estimates. Our methods combine information from an available travel survey, estimated travel routes, and concentration data from air pollution modeling results. We address the following questions through this work: How are population activities distributed spatiotemporally in the study domain? How are exposures distributed among population groups in the study domain? What is the strength and direction of disparities between groups? Does urban form influence the strength of exposures and their social distribution? Which factors are most influential? Are findings robust to uncertainties in exposure estimation associated with the representation of exposure location? How much does the representation of spatiotemporal activity location impact exposure estimates? Are the errors associated with exposure estimation different for different population subgroups? Methods and findings on these questions are detailed below.

## Methods

### Study area and pollutant focus

**Fig. 1:**
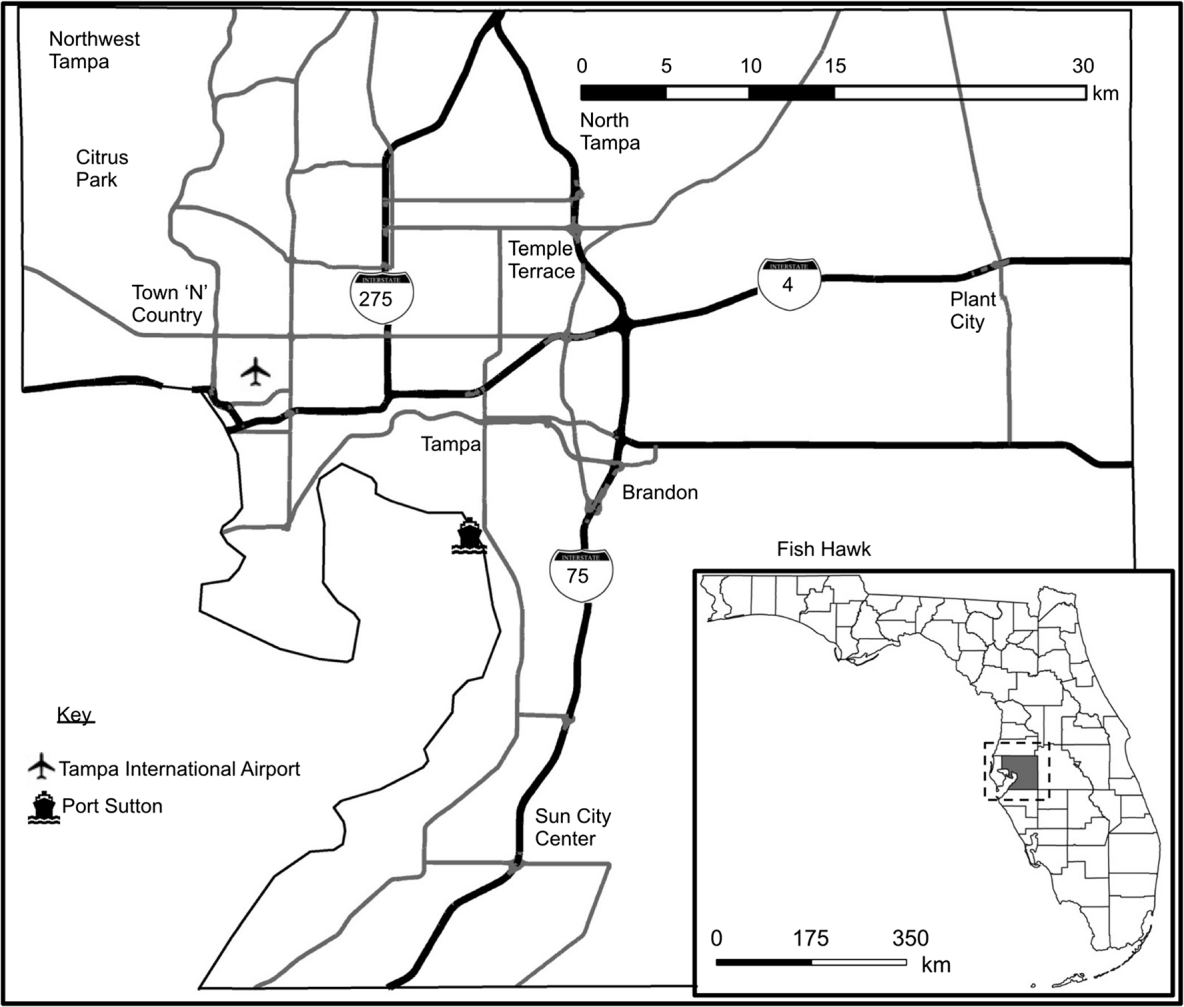
The study area of Hillsborough County, FL. The *inset* provides the location of the study area within the state of Florida

Hillsborough County, FL, shown in Fig. 1, is the area of study. The area contains a diverse mix of air pollutant emission sources, including an extensive highway network. Further, it has undergone considerable urban sprawl during the past few decades; in 2000, Smart Growth America ranked it as the 22nd most sprawled metropolitan area (out of 83 with populations over a half million) (Ewing et al. 2002). In 2012, the Texas Transportation Institute ranked Tampa-St. Petersburg as 30th for congestion (yearly delay per commuter) (Schrank et al. 2012), with automobiles as the primary mode of personal transportation. Regarding measured air quality, ozone levels exceed the National Ambient Air Quality Standard a few times most years, while particle levels are close to the 24-h standard. The American Lung Association grades the county’s air quality as F for ozone and C for particulate matter (American Lung Association 2011). Further, the county is interesting for social equality reasons, as its population is relatively diverse and somewhat residentially segregated (Stuart et al. 2009).

The pollutant focus of this paper is oxides of nitrogen (NO_x_), which is the sum of nitrogen monoxide (NO) and nitrogen dioxide (NO_2_, a US criteria air pollutant with an established standard level). Although, levels of NO_2_ measured by regulatory networks rarely exceed the national standard, NO_x_ is a precursor to both ozone and fine particles. Further, it is a common urban pollutant that has been associated with respiratory responses for susceptible individuals, particularly children, even at levels below the National Ambient Air Quality Standard (US Environmental Protection Agency 2008). Studies have linked exposure to oxides of nitrogen with cardiovascular and respiratory mortality (Faustini et al. 2014), gestational diabetes and preeclampsia (Malmqvist et al. 2013), diabetes mellitus and hypertension (Coogan et al. 2012), and incidence of asthma (Anderson et al. 2013). NO_x_ is also a recognized surrogate in health outcomes analyses for the complex mix of traffic pollution (HEI Panel on the Health Effects of Traffic-Related Air Pollution 2010).

### Estimation of spatiotemporal human activity-travel patterns

Human activity-travel patterns representing the study area were estimated using data from the National Household Travel Survey (NHTS). The periodic survey characterizes the daily travel behavior of Americans (Federal Highway Administration 2009). Data are collected on all out-of-home trips taken over approximately a 24-h period for individuals sampled by the survey. The data collected include the purpose of each trip (work, shopping, recreation, etc.), trip start and end times, travel times, travel distances for each trip, and the geocoded locations of activities. Sociodemographic characteristics (including age, race/ethnicity, household income, household size, and neighborhood urbanicity) of those surveyed are also collected. Here, we used the data from the 2009 survey to characterize spatiotemporal locations of daily activity and travel in Hillsborough County, FL.

The National Household Travel Survey sample for Hillsborough County includes daily activity records for 1,582 persons from 804 households. Prior to use, we filtered the sample to exclude daily activity records that were inconsistent or had missing information. We also excluded records that contained travel outside of the county boundaries (beyond which detailed NO_x_ concentrations were not available). For a few records, it was necessary to pare the data to exactly 24 h (beginning at 12:00 a.m.). The resulting sample consisted of 1,224 daily activity records, including 239 with no travel away from the residence location on the survey day.

To estimate the locations of daily activities in time and space for the county sample, we first extracted data from each individual 24-h activity record (a person-day). Specifically, we extracted the geocoded residence location (latitude, longitude), origin and destination locations for each trip, trip start times, and dwell times (time spent at the activity location) using SPSS (version 20.0, IBM Corp. Armonk, NY). Since the National Household Travel Survey does not record information on travel path, we estimated the route of travel for each trip. Specifically, we used the Network Analyst tool in ArcGIS (version 10.0, ESRI, Redlands, CA) to select the shortest time path between each trip’s origin and destination, based on roadway link times and a network shape file (NAVTEQ 2010). Travel times for each link were calculated using link lengths and link free-flow speeds provided with the network data. Spatial location coordinates (latitude, longitude) along each trip path were extracted at a discrete interval of 100 m of path length using the ET GeoWizards tool (version 10.2, ET Spatial Techniques, Faerie Glen, South Africa). The temporal location coordinate (time of day) for each discrete spatial location was estimated by adjusting the time on each link by the ratio of the total trip time from the survey data to that from the link time estimate. We then combined the trip path location data to create a highly resolved sequential spatiotemporal record of estimated activity location for each person-day in the filtered county sample.

### Estimation of diurnal pollutant concentrations at activity locations

To estimate pollutant exposures for the study sample, we used ambient NO_x_ results from our previous dynamic CALPUFF air pollution dispersion modeling for the study area. Details of the modeling methods, results, and evaluation are provided in Yu and Stuart (2013). In essence, concentrations were estimated using detailed emissions, including link-level roadway emissions, and meteorological data for 8,760 h (all hours of 2002) for the study area. The results provide estimated concentrations on a receptor grid with 1-km spatial resolution for Hillsborough County. For matching with the daily activity-travel records here, we estimated the diurnal cycle of the spatial distribution of NO_x_ concentration from the model results by averaging the hourly modeled concentration results at each receptor over each hour of the day.

### Estimation of daily exposure concentration and exposure error

One goal of this work was to investigate the impact of activity-travel patterns on exposure estimates. To do this, we calculated and compared daily exposure concentrations for each person-day using two methods. Both methods estimate the time-weighted exposure concentration, *C* = (1 / *T*) ∫*cdt*, where *c* is the instantaneous pollutant concentration at an exposure location, *dt* is the instantaneous time interval of exposure, and *T* is the total exposure averaging period, which equals ∫*dt* (24 h for the person-day records here).

The first method uses only the residence location to estimate daily exposure concentration for each person-day. We call this the residence-based exposure concentration (*C*_*R*_); it represents conventional exposure concentration estimation using only residence address information. Since the spatial location of exposure does not change with this approach, the discretized exposure concentration during each person-day varies in time only, not in space. Using the ArcGIS intersect tool, we extracted concentrations from the 24 dispersion modeling concentration maps (each representing 1 h of the day with 1-km spatial resolution), resulting in ambient concentrations (*c*_*τ*_) for each hour of the day (*Δt*_τ_, equal to 1 h) at each residence location. We then numerically integrated these data using time weighting in SPSS to estimate the daily residence-based exposure *C*_*R*_ = (∑*c*_τ_ *Δt*_τ_) / *T* for each person-day in the study sample.

Second, we estimated daily exposure concentrations by matching the spatiotemporal locations in each person-day activity-travel record with modeled concentration at those locations; we call this the activity-based exposure concentration estimate (*C*_*A*_). Specifically, we extracted concentrations from the modeled data for each discrete location along each person-day activity-travel path. This results in ambient pollutant concentration (*c*_*σ*_) and time spent (*Δt*_*σ*_) for each discretized spatiotemporal activity-travel path location, *σ* = (latitude, longitude, time). Note that concentration for the same hour of day changes due to movement in space. The daily activity-based exposure concentration was then numerically estimated as *C*_*A*_ = (*∑c*_*σ*_ *Δt*_*σ*_) / *T* for each person-day in the study sample. For explanatory analyses, we also estimated exposures, *E*_*A*_ = ∑*c*_*σ*_ *Δt*_*σ*_, for subdaily periods.

To compare the two measures of exposure concentration, we calculated the percent difference between the activity-based and residence-based exposure concentration as (*C*_*A*_−*C*_*R*_) / *C*_*A*_ for each person-day in the sample. We call this the exposure error, as it estimates the error associated with using residence location only to calculate exposure. Frequency distributions for the study sample of daily exposure concentration (estimated using both methods) and of exposure error were compared to describe differences. A paired sample *t* test was used to quantify the significance of differences in the means for each sample distribution. Finally, we calculated bias factors to quantify the potential bias in relative risk estimates (based on simple linear models) due to use of the residence-based exposure estimate, following the method outlined by Setton et al. (2011).

### Analysis of exposure distributions and inequality

A second goal was to characterize disparities between groups in their activity-based exposure concentration and in potential exposure error, including identification of factors impacting both. To do this, we first categorized daily exposure concentrations and exposure errors by population subgroup. We focused on subgroup types representing characteristics that have previously been found to experience exposure disparities or air pollution susceptibility. Specifically, the person-day exposure concentration estimates were categorized by age (5–18, 19–45, 46–65, and greater than 65 years), race/ethnicity (Asian, white, Hispanic, and black), and household income/poverty (below poverty, above poverty to below $75,000, and above $75,000). Age less than 5 could not be considered, as no survey data are available for this category. To define the poverty threshold, we use the 2009 federal poverty guidelines that are based on household size (Department of Health and Human Services 2009). The above $75,000 threshold was chosen to capture approximately the highest third of the income distribution in the study area. After categorization, group frequency distribution summary statistics (e.g., mean, percentiles) were calculated and compared. To measure the significance of differences between groups of the same type, we used 95 % confidence intervals around each mean and performed one-way ANOVA, followed by post hoc Games-Howell testing (Hayes 2005). A similar analysis was performed for differences in exposure errors between groups.

To investigate impacts of urban design and activity factors on exposure and exposure disparities, we performed a few additional analyses. As a proxy for urban design, we first categorized exposure concentrations by the urbanicity of the residence location (urban, suburban, second city, rural), as provided with the survey data. (Claritas Inc. 2004 provides urbanicity category definitions; we use the term rural for the town and country category, to avoid confusion with the proper name—Town “N” Country—of a region in the study area. See Fig. 1.) Distributions of activity-based exposure (concentration × time) were also compared between different activity location types to explore the contribution of activity-location type to exposures. Specifically, activity location types were divided into three categories—at-residence, nonresidential, and in-travel. Exposures were also compared against daily travel time. Similar analyses were performed for exposure error. Finally, we performed a multivariate linear regression analysis to assess the impact of urban design and activity factors on exposure concentration. Specifically, we used a hierarchical stepwise approach, in which the sociodemographic predictors (gender, age, racioethnicity), followed by the income categories, were introduced first, to control for their impacts on exposure concentration. The urbanicity categories, followed by the activity time variable, were entered subsequently. All predictor variables were introduced into the model as categorical binary variables (e.g., male/female, black/nonblack), except the time variable, which was introduced as a continuous variable. A 95 % confidence (*p* < 0.05) statistical significance criteria for each predictor variable was used to discard or retain variables at each modeling step. All statistical analyses were performed in SPSS.

## Results and discussion

### Distributions of human activity in the Tampa area

Table 1 provides a summary of the average temporal distributions of activity types observed by the 2009 National Household Travel Survey for the filtered study sample in Hillsborough County. Time activity data are also provided from two well-known historical human activity surveys used for exposure analysis, the National Human Activity Pattern Survey (NHAPS) (Klepeis et al. 2001) and the Canadian Human Activity Pattern Survey (CHAPS) (Leech et al. 1996). As in the historical surveys, residents in the study sample spent the majority of their time at home (about 80 %), although the percentage of time spent at home is about 13 % higher here. The order (from highest to lowest percentage of time spent) of activity location types is also the same here as in the NHAPS and CHAPS. However, the quantitative distribution of time is somewhat different; the population sampled here spent more time on average at work and less time travelling, at meals, and at other activities. Some of these differences may be due to differences in spatial scale, geography, and demographics. Specifically, the NHTS results are for Hillsborough County, FL, while the NHAPS and CHAPS results are for the entire USA and Canada, respectively. Further, Florida is a state with large elderly population, which may contribute to more time spent at home. Finally, different definitions of the activity-type categories between the surveys could also have led to some differences.

**Table 1:** Average time spent per day by activity location type

	This work^a^		NHAPS^b^	CHAPS^c^
Activity type^d^	Minutes	Percentage	Percentage	Percentage
Home	1,151	80	67	67
Other	116	8.0	19	20
Work	98	6.8	5.9	6.0
Travel	62	4.3	5.7	5.3
Meals	13	0.9	1.9	1.8

^a^The filtered sample from the 2009 National Household Travel Survey (NHTS) for Hillsborough County

^b^National Human Activity Pattern Survey (Klepeis et al. 2001)

^c^Canadian Human Activity Pattern Survey (Leech et al. 2002)

^d^The following specific categories from each study were included under each label. Home refers to the NHTS home category and the NHAPS and CHAPS categories of indoor at home and outdoor at home. Work refers to the NHTS work category and the NHAPS and CHAPS office/factory category. Travel refers to the NHTS categories of travel and transport someone and the NHAPS and CHAPS categories of in vehicles and near vehicles-outside. Meals refers to the NHTS meals category and the NHAPS and CHAPS bar/restaurant category. Other refers to the NHTS categories of school/daycare/religious activity and medical/dental services, shopping/errands, family personal/business obligations, social/recreational activities, and other categories and the NHAPS and CHAPS categories of school/public building, indoors-other, outdoors-other, and mall/store

**Fig. 2:**
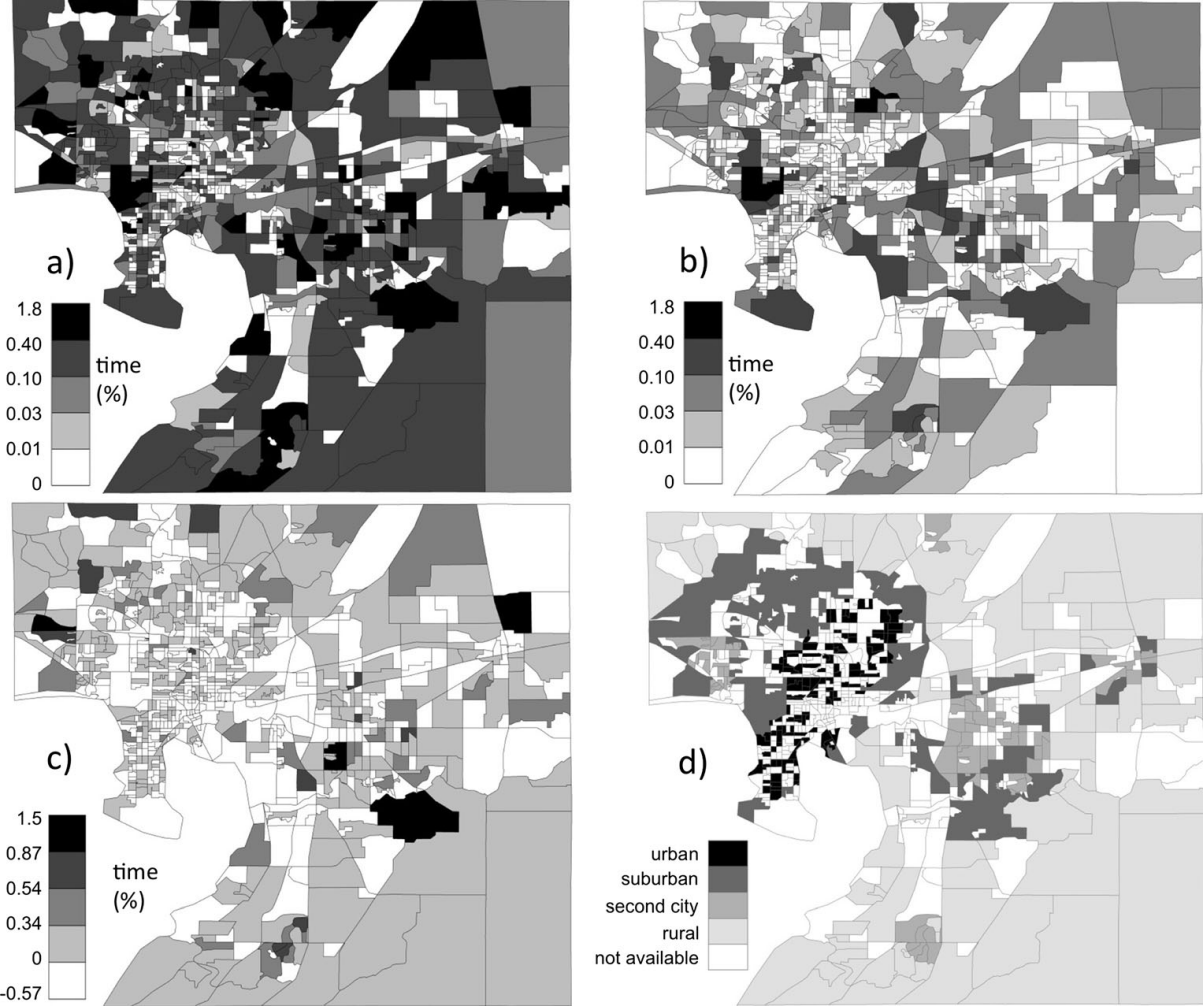
The spatial distribution of sample population activity time (% of time spent) and urbanicity in the study area. **a** % of total time spent in all activity types within the block group, **b** % of total time spent in nonresidential activities within the block group, **c** difference (%) between residential and nonresidential activity times spent in each block group, and **d** urbanicity category of the block group

Figure 2 provides the spatial distribution of activity time from the study sample (the percentage of total time spent in each block group area (subplots a–c), along with urbanicity (subplot d) of each block group). To our knowledge, this presentation of a spatially distributed activity time density map applied to exposure analysis is novel. Subplot d indicates that urbanicity generally decreases from central Tampa, surrounded by suburbs (including Citrus Park and Temple Terrace). A few pockets classified as second city areas (Sun City Center, Brandon, Plant City, New Tampa, and Town “N” Country) are farther from central Tampa and are surrounded by areas classified as the rural urbanicity category. The block group areas in the largest time density category (with at least 0.4 % of the total time in the sample) are located in areas that are categorized as rural (e.g., Fish Hawk), suburban, and some second city locations (e.g., in Brandon and Sun City Center) and largely correspond to the areas with the highest percentage of residential activity time (not shown). The block groups with the highest densities of nonresidential time are largely special locations (Tampa International Airport, the University of South Florida) or, for the second highest category (containing from 0.1 to 0.4 % of total time in the sample), in rural, second city, or suburban locations. As a whole, the population of the Hillsborough County sample spent little time in urban block groups, though the time densities are larger for nonresidential activities.

### Diurnally varying spatial distributions of NO_x_ concentration

**Fig. 3:**
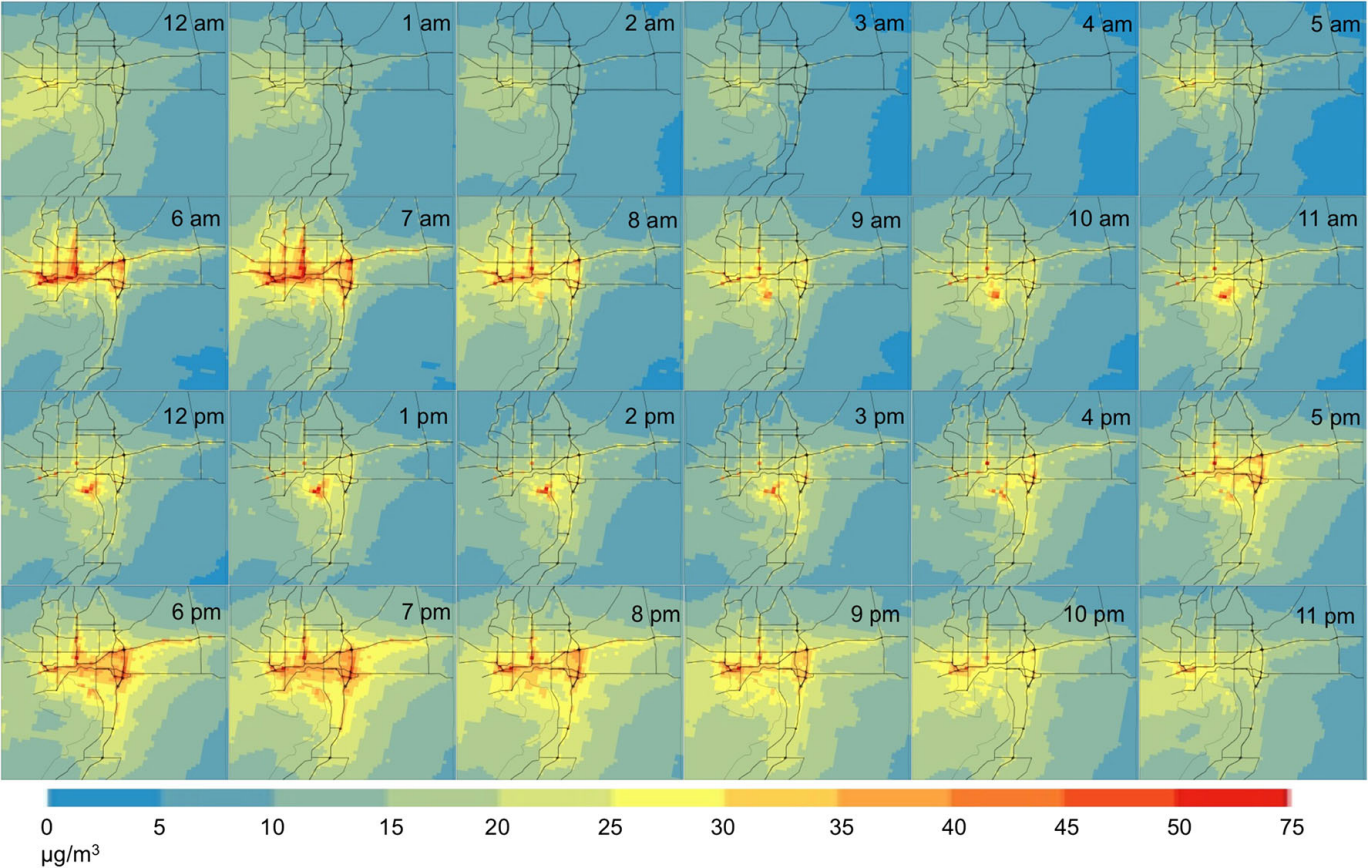
The estimated diurnal cycle of hourly average ambient NO_X_ concentrations (μg/m^3^) in the study area, from dispersion modeling results

The average diurnal cycle of modeled NO_x_ concentration for the study area is shown in Fig. 3. See Yu and Stuart (2013) for a detailed discussion of the spatial distribution of concentrations in the study area and results from evaluation of model performance. For our purposes here, note that for many hours of the day, the concentrations are highest along the major roadways in the area with a broad peak apparent over central Tampa and near the Tampa International Airport. A high concentration area is also often visible near a major port facility (Port Sutton) to the south of downtown. Diurnally, concentrations exhibit morning (6:00 a.m. to 8:00 a.m.) and evening (5:00 p.m. to 9:00 p.m.) peaks, consistent with increased NO_x_ emissions from traffic during commute hours. The evening peak is more spread out in time than the morning peak, consistent with both a larger meteorological mixing height in the evening and typical commute behaviors; specifically, the morning commute is known to be largely driven by work-related activities, while the evening commute may include maintenance, social, recreational, and other activities (Jou and Mahmassani 1997; Kim et al. 2008). A detailed evaluation of modeled estimates against measured data is provided in Yu and Stuart (2013).

### Daily time-weighted activity-based exposure concentrations and their social distribution

**Fig. 4:**
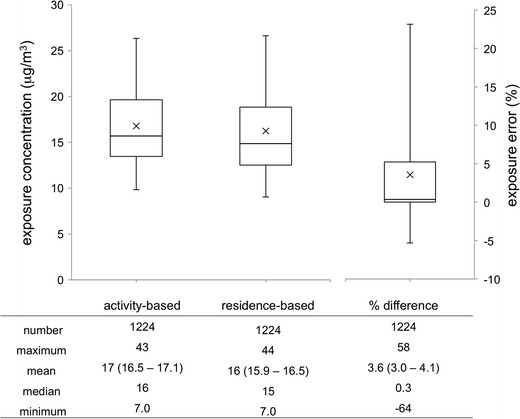
Cumulative distributions for the activity-based daily exposure concentration (*left side*), residence-based daily exposure concentration (*middle*), and daily exposure error between the two as a percent difference, (*C*_A_−*C*_R_) / *C*_A_ (*right side*). The *box plot whiskers* indicate the 5th and 95th percentile values, while *cross* indicates the mean value. Summary statistics are provided below each box plot; 95 % confidence intervals around each mean are in *parentheses*

The cumulative distribution of estimated daily (24 h) activity-based NO_x_ exposure concentration is shown in Fig. 4 (left side). The mean exposure concentration for the study sample is 17 μg/m^3^, with values for individual person-day records ranging from 7.0 to 43 μg/m^3^. Using a typical fraction of NO_2_ in NO_x_ estimated for the Tampa area of 0.8 (Poor 2008), the values found here roughly correspond to daily NO_2_ exposure concentrations of 7.4 and 18 ppbv for the sample mean and maximum, respectively. Although these values are on the low end of 24-h NO_2_ exposure concentrations measured elsewhere (e.g., Delfino et al. 2008; Kim et al. 2006), they are in the range of 24-h average NO_2_ exposure concentrations that have been found to be associated with a variety of respiratory-related health outcomes (US Environmental Protection Agency 2008).

**Fig. 5:**
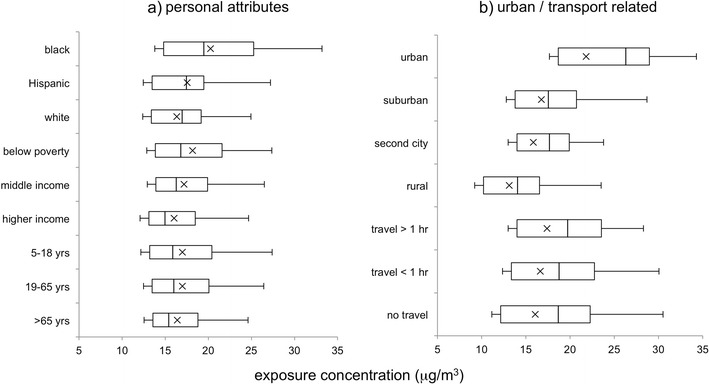
Cumulative distributions of daily activity-based exposure concentration for population subgroups related to **a** personal attributes and **b** urban characteristics. Category definitions are provided in the text. Note that the racioethnic subgroup populations are not exclusive, populations have overlapping individuals

The cumulative distributions of the activity-based daily NO_x_ exposure concentrations for a few subgroups are provided in Fig. 5. Distribution statistics are provided in Table 2. Apparent differences in exposure concentrations among subgroups in the racioethnic and income categories are seen. Among the racioethnic groups, estimated mean daily exposure concentration is highest for the black group (20 μg/m^3^), followed by the Hispanic group; mean exposures were lowest for whites (16 μg/m^3^). Results for the Asian subgroup are not shown due to the small sample size (14 person-days). Although within-group variations increase with increasing group mean concentrations, the 95 % confidence intervals around the means for the black and white categories are far apart, and one-way ANOVA with post hoc Games-Howell testing also indicated high significance for the difference (*p* = 6 × 10^−8^). Differences between the other categories were not significant, as the confidence intervals overlap. Among the income categories, the mean daily exposure concentration was highest for the group characterized by household income below the poverty level (18 μg/m^3^). This value is slightly lower than that estimated for the black group. Mean exposure concentration decreases with the income category, to 16 μg/m^3^ for the group characterized by higher incomes (household annual incomes above $75,000). The confidence intervals and post hoc testing indicate statistically significant differences between means for the below poverty versus highest-income group, and between the two above poverty groups, but not between the below poverty versus middle-income group. Differences in mean exposure among the age-based groups are not apparent.

**Table 2:** Group distribution statistics for daily activity-based exposure concentration and exposure error

		Exposure concentration (μg/m^3^)				Exposure error (%)			
Group	Values of *n*	Mean	Confidence interval	Min	Max	Mean	Confidence interval	Min	Max
Race/ethnicity^a^	1,173								
Black	115	20	(19.0–21.5)	8.5	43	1	(−1.1–3.3)	−64	34
Hispanic	29	18	(15.9–19.2)	11	28	5	(1.3–9.6)	−8.9	45
White	1,029	16	(16.0–16.6)	7.0	41	4	(3.3–4.4)	−32	58
Income^b^	1,131								
Below poverty	137	18	(17.1–19.2)	7.4	43	1	(−0.6–2.6)	−64	45
Middle income	577	17	(16.8–17.6)	7.0	41	3	(1.9–3.5)	−52	53
Higher income	417	16	(15.6–16.5)	7.4	32	6	(4.6–6.5)	−31	58
Age	1,224								
5–18 years	148	17	(16.1–17.9)	8.5	29	3	(0.6–4.6)	−64	53
19–65 years	665	17	(16.6–17.4)	7.0	41	5	(4.2–6.0)	−48	58
Over 65 years	411	16	(15.9–16.9)	7.4	43	1	(1.0–1.8)	−17	23
Urbanicity	1,224								
Urban	267	22	(21.2–22.4)	12	43	0	(−1.3–0.9)	−64	25
Suburban	387	17	(16.3–17.2)	10	35	4	(3.3–5.2)	−31	42
Second city	287	16	(15.5–16.2)	8.8	25	4	(3.1–4.9)	−31	38
Rural	283	13	(12.6–13.6)	7.0	27	6	(4.3–7.1)	−17	58
Daily travel time	1,224								
More than 60 min	452	17	(17.0–17.8)	8.5	41	8	(6.5–8.8)	−32	58
Up to 60 min	533	17	(16.2–17.1)	7.6	43	2	(1.0–2.4)	−64	42
No travel	239	16	(15.3–16.7)	7.0	35	0	(0–0)	0	0

^a^Racioethnic labels used here are shortened forms of the race and origin category labels used by the US Census. Category descriptions are available at www.census.gov. Note that placement in a category is by self-selection, and individuals may be categorized in multiple or no categories

^b^The below poverty, middle-income, and higher-income labels refer to households with income below the poverty threshold, above the poverty threshold but less than $75,000 and $75,000 or above

Differences in daily activity-based exposure concentrations observed here between the racioethnic groups are consistent with our previous studies that have estimated exposures in the Tampa area using only residence location (Stuart et al. 2009; Yu and Stuart 2013) or school location (Stuart and Zeager 2011). Specifically, we found greater exposures for the black, Hispanic, and low-income (below poverty) groups than the white and higher-income groups, respectively. Hence, regardless of the use of individual-level activity information in the exposure estimation, the qualitative direction of the disparities found for the Tampa area appears to be robust.

Furthermore, results are consistent with other findings from the study area and elsewhere. Specifically, in a study of the Tampa area using 1999 National-scale Air Toxics Assessment concentration data and the population distributions from the 2000 US census, Chakraborty (2009) found that the black, Hispanic, and below poverty groups are subject to disproportionate cancer risks and respiratory hazards, while no conclusive inequities were found for individuals above 65 years (Chakraborty 2009). Overall, results here contribute to the body of literature across localities in the USA and elsewhere (Green et al. 2004; Houston et al. 2004; Linder et al. 2008; Marshall et al. 2006; Marshall 2008; Mitchell and Dorling 2003; O’Neill et al. 2003; Pearce et al. 2006), largely finding typically higher exposures to primary pollutants for socially and economically disadvantaged groups, with some exceptions (Buzzelli and Jerrett 2007) and reverse finding for secondary pollutants (Marshall 2008).

It is worthy of mention that the use of spatiotemporal activity information in this study did not change the relative ranking of mean disparities between racioethnic versus income groups. The mean difference between the black and white category was larger than the difference between the below poverty and highest-income group; blacks had the highest estimated average exposure of all racioethnic or income groups. However, this result is complicated by the results of regression analysis (discussed below), for which income below poverty was associated with a larger independent increase in exposure concentration (1.7 μg/m^3^), than being black (versus nonblack, 1.2 μg/m^3^). However, the comparative difference in group mean disparities found here is consistent with results from other study areas. Specifically, in a study in southern California, Marshall et al. (2006) found that exposure levels differed more among ethnic groups than between high- and low-income households, while Clark et al. (2014) found a similar result through a national-level analysis. We note that there are many aspects of social disadvantage that are not captured by race, ethnicity, or income alone. Further, it is well established that there are interactions between factors that affect exposure disparities (e.g., Apelberg et al. 2005; Perlin et al. 2001), with many studies in the air pollution field now using multifactor indices that can also include education, occupation, employment status, family size, and home ownership (e.g., Forastiere et al. 2007).

Although differences in daily exposure concentrations are evident in our results, their importance to health outcomes is not necessarily clear. To explore the potential importance, we applied literature estimates of increased risk (primarily as reported by the US Environmental Protection Agency 2008) to estimate possible health impacts. Neuberger et al. (2007) found a 2.9 % increase in risk of total mortality associated with a 10 μg/m^3^ increase in 24-h mean NO_2_ concentrations. Applying this to the differences in group means found here would suggest an increased risk of 1 % for blacks versus whites (on average) and an increased risk of 0.5 % for those living in poverty versus in households with annual incomes over $75,000 (on average). Even higher differences in health risks may be present between groups, when considering susceptible people, such as children and the elderly. Application of the 61.3 % increased risk for cough incidence per 20 ppbv increase in 24-h NO_2_ concentration found by Schwartz et al. (1994) in a study of children would result in an approximately 5 % excess risk for black compared to white children here, on average. Similarly, applying the ratio of 6.8 % increased risk of all respiratory hospitalizations per 20 ppbv increase in daily NO_2_ concentrations found in a study by Fung et al. (2006) of adults aged 65 and older suggests a 0.6 % higher risk for elderly blacks compared to elderly whites here, on average. Note that for any individual, the comparative risks may be higher or lower due to individual risk factors (smoking, diet, exercise, occupation, access to health care, etc.) (Dockery et al. 1993; Pope et al. 2002). Additionally, since differences in harmful health effects have been found even when differences in exposures are not clear (Deguen and Zmirou-Navier 2010), small differences in exposures between groups may be important.

Overall, our results suggest that to attain the policy goal of reducing disparities in health outcomes (Healthy People 2020 and US Department of Health and Human Services 2010), interventions that reduce existent disparities in exposure between socioeconomic groups may be helpful. Further, the methods used here provide an approach for estimating activity-based exposures specific to individual person-days, but for a large sample. This could be helpful for the study of factors affecting population health outcomes and for estimation of expected risks, without the intractably large costs of personal exposure concentration sampling for a large population.

### Urban form, activity, and exposure relationships

We are interested in understanding how factors related to urban form may impact the magnitude of exposures and their social distribution in the Tampa area. Figure 5b provides the distributions of estimated daily activity-based exposure concentrations categorized by the urbanicity of residence location, with statistics provided in Table 2. Substantial differences in NO_x_ exposure concentrations are seen between residence urbanicity types. The highest mean daily exposure concentration (22 μg/m^3^) was found for records with urban residence location, while that for records with rural residence location was 40 % lower (13 μg/m^3^). Mean exposures were intermediate for the suburban and second city categories. The confidence intervals and post hoc testing indicate that all differences between the category means are significant. The largest difference in means between urban versus rural residence urbanicity categories (9 μg/m^3^) is also more than twice as large as the largest difference among the social categories discussed above (4 μg/m^3^ for the black versus white subgroup mean difference). Hence, residence urbanicity likely influences exposure and its social distribution among groups. This is broadly consistent with results of previous studies comparing exposures for populations in urban versus rural areas. For example, in the EXPOLIS-Helsinki study, individuals living downtown had 23 % higher exposures than suburban residents (Rotko et al. 2001). Similarly, in a study of school children, Rijnders et al. (2001) found that both outdoor and personal NO_2_ exposures increased with the level of urbanicity (and traffic density), with a mean difference in personal exposures for the highest versus lowest urbanicity category of 14.6 μg/m^3^.

Results of the multivariate linear regression analysis (Table 3) also indicate that urbanicity was the strongest predictor of exposure concentrations (with the highest coefficient value and *t* statistic) among the factors studied. A model using only the urbanicity variables as predictors (not shown) captures about 35 % of variance in the individual exposures, a substantial portion of the total model variability captured in the more complex model shown. Consideration of interaction terms between the sociodemographic and urbanicity variables provides further insight on the influence of residence urbanicity on the disparities in exposure found above between sociodemographic groups. Specifically, interaction terms urban × black, suburban × black, and suburban × below poverty, all had significant *t* statistics (significance values of 0.002, 0.005, and 0.004, respectively) and high coefficients (3.2, 3.1, and 2.5 μg/m^3^, respectively) when added to the base model shown. Furthermore, with the interaction terms added, the black explanatory variable (which now represents blacks living in second city and rural regions) was no longer significant, and the below poverty variable had substantially reduced significance (0.04 with a reduced coefficient value of 0.9 μg/m^3^). Additional comparisons of subgroup exposures (not shown) also indicate that, for those living in second city and rural regions, the difference in the mean exposure for blacks versus others is not significant. That is, the exposure disparity (higher group mean daily exposure concentration) found here for the black group on average, above, is due to both higher exposures for the urban and suburban black population and higher residence urbanicity for the black population in the study area. Residence urbanicity classification also explains some of the disparity between economic groups, but the result is more complicated, as an urban below poverty interaction term was not found to contribute significantly, but the suburban below poverty term was.

**Table 3:** Linear regression model for activity-based exposure concentration

Explanatory variable	Coefficient^a^ (*β*_i_, *γ*_i_)	*T* statistic	Significance
Constant^b^ (β_0_)	11.5	35	3e-180
Black	1.2	2.9	3e-3
Below poverty	1.7	4.3	2e-5
Middle income^c^	1.2	4.7	2e-6
Urban	8.3	23	1e-98
Suburban	3.4	11	8e-25
Second city	2.6	7.5	1e-13
Time away from residence	0.2	6.8	1e-11
Goodness of fit			
*R* ^2^	0.40		
Adjusted *R* ^2^	0.39		
Number of cases	1,120		

^a^The regression model is *C*_A_ (μg/m^3^) = *β*_0_ + *β*_1_ *X*_c1_ + *β*_2_ *X*_c2_ + …*γ*_1_ *X*_t1_ + *γ*_2_ *X*_t2_ + … + *ε*, where *X*_ci_ ∈ {1,0} are the binary variables and *X*_ti_ are the continuous variables. Only the time variable was entered as a continuous variable (with units of hours). *β*_i_ have units of μg/m^3^, *γ*_i_ have units of (μg/m^3^)·h

^b^The constant concentration represents exposures for people who are nonblack with incomes over $75,000, who live in town/rural areas and did not travel on the sample day

^c^Middle income refers to households with income above the poverty threshold but less than $75,000

**Fig. 6:**
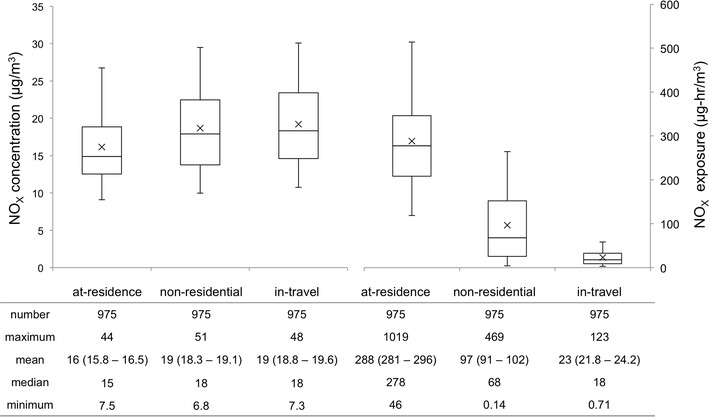
Cumulative distributions of time-weighted NO_X_ concentration (μg/m^3^) (*left-side*) and NO_X_ exposure (μg/m3)·h (*right-side*) by activity type location for all sampled daily records including some activity away from residence. Summary statistics are provided below each *box plot*; 95 % confidence intervals around each mean are in *parentheses*

Although it is known that residence urbanicity is associated with increased exposure, the reasons for this are not well understood. One contributing reason that has been explored extensively is the presence of higher concentrations of pollutants in urban versus rural areas. We can clearly see in comparing Figs. 3 and 2d that NO_x_ concentration is generally higher in urban versus rural areas throughout the day. However, we look here at the additional role of activity, with a focus on travel activity. Figure 6 provides NO_x_ concentration and exposure distributions categorized by activity-location types (at-residence, nonresidential, and in-travel) for the subsample (*n* = 975) of person-day records that included some activity in each category on the survey day. The mean (time-weighted) concentration is highest (19 μg/m^3^) for the in-travel activity category and lowest (16 μg/m^3^) for the at-residence category. That is, ambient concentrations were higher at the locations of nonresidential and travel activities (at least during the times when our sample population was located there). However, mean exposures (μg/m3)·h are lower for travel and nonresidential activities, as less time is spent in these activities (see Table 1). Overall, the group mean daily exposure concentration increases for those who travel more (Fig. 5b). Confidence intervals for the categorical means (Table 2) indicate significantly different group mean exposure concentrations for daily activity records with more than 60 min of travel time versus those with no travel. Our multivariate linear regression (Table 3) also indicates a small increase in exposure concentration with increased daily time away from the residence location (travel time plus time at nonresidential locations), with concentrations increasing by 0.2 μg/m^3^ per hour of total daily time. Hence, although residence location remains a better predictor of daily exposure concentration than does time away from the residence (or time travelling), these activity times may play a role.

These results are consistent with those of other recent studies indicating the importance of exposures during travel. de Nazelle et al. (2013) found that travel activities contributed 24 % of the total daily intake of NO_2_. Dons et al. (2012, 2011) found that transport time accounted for 21 % of black carbon exposures and identified transport activity as a primary reason for differences in exposure between family members. Zhang and Batterman (2013) also recently found that increased traffic congestion led to greater population health risks; for the on-road population, this was due in part to increased transport times. We found the contribution of time in travel to be less for our study area, accounting for 6 % of the total daily exposure on average, but time at nonresidential locations accounted for 24 %.

From an exposure mitigation perspective, it is known that higher concentrations of many pollutants in urban areas are largely due to the proximity and spatial concentration of air pollution sources in urban areas, including car exhaust on congested roadways, combustion emissions from home heating, and nearby industrial emissions. Hence, mitigation policies have focused on reducing emissions from sources (e.g., through engineering control technologies). However, reduction in exposures requires reduction in emissions at a rate outpacing economic and population growth, which has proved difficult to sustain. Another popular strategy has been urban design that displaces sources away from where people live via urban planning and zoning policies (South Coast Air Quality Management District 2005). However, this strategy has resulted in collocation of sources with socially disadvantaged population groups who cannot afford to live in less polluted areas (Perlin et al. 2001; Pulido 2000), and with increases in emission-producing travel necessary for people to access their homes, places of employment, and services.

Hence, “smart growth” urban design strategies are now being promoted as potentially mitigating exposures (Office of Sustainable Communities et al. 2013). Previous work has suggested that high-density urban growth can potentially help in reducing the vehicle miles travelled and the overall emissions (Hankey and Marshall 2010; Stone et al. 2009). However, simply applying land use intensification (or densification) strategies without making modifications to the existing transportation infrastructure might increase congestion and lead to higher concentrations in urban areas (Farber et al. 2009). This could also exacerbate social disparities in exposures, as many disadvantaged groups disproportionately live in more dense urban areas (Baum et al. 1999). This underscores the need for caution in implementing high-density developments alone. However, another informative viewpoint may be differences in activity behavior that place people in spatiotemporal locations of high concentrations. Particularly interesting from a policy viewpoint are activity behaviors that are impacted by civic infrastructure. In this study, we found that average concentrations were higher in travel and nonresidential activities, and estimated daily exposures were higher for those who travel more. Hence, a focus on civic infrastructure that reduces time travelling (and other nonresidential activities) as well as emissions at those locations may be warranted. Implementation of transit infrastructure is one such approach, as it can reduce congestion (with concomitant reductions in emissions) and can reduce the time spent travelling on congested roadways. However, cost-competitive transit infrastructure requires high-density development (Kenworthy and Laube 1999).

### Exposure error

The cumulative distributions of estimated residence-based daily NO_x_ exposure concentration and exposure error (due to the use of a residence-based versus activity-based approach) are shown in Fig. 4. Overall, we found the mean exposure error [(*C*_*A*_−*C*_*R*_) / *C*_*A*_] to be 3.6 %, with a range of −64 to 58 %. Additionally, for the majority of the sample (56 %), the error is positive (the activity-based exposure estimate is larger than the residence-based estimate). There is a small amount of overlap in the confidence intervals around each mean, though a paired sample *t* test suggests statistically significant differences (*p* = 3e-22). Additionally, for the subsample of person-day records (*n* = 985) that included at least some travel away from the residence on the survey day, the mean error is slightly increased (4.4 %). The calculated bias factors for the full sample and for travelling subsample were 0.85 and 0.82, respectively, indicating that in a health impact study using residence-based daily exposure estimates, the relative risk may be underestimated by 15 or 18 % for the travelling sample.

Mean exposure bias (or error) values observed in our study are consistent with previously reported results and suggest the importance of consideration of activity-travel patterns for exposure estimation. For Metro Vancouver, Setton et al. (2011) have reported an exposure bias for residence-based versus activity-based exposure estimates of 0.70 to 0.84 for NO_2_ (depending on the method used for concentration interpolation). Further, in a study of Flanders and Brussels, Dhondt et al. (2012) found small but significant differences between the mean dynamic (i.e., activity-based) exposures and residential exposures (21.6 versus 20.98 μg/m^3^), with a resulting exposure error of 2.9 %. Similarly, in a Belgian study, Dons et al. (2011) found that time-activity patterns could account for approximately 30 % of weekly personal mean exposure differences between a worker and a homemaker from the same household. While their study does not consider exposure error explicitly, their findings underscore the importance of time-activity patterns and their impact on exposures.

Our results suggest that a residence-based approach likely underestimates exposures for a large proportion of the population, resulting in underestimated risks of health impacts of air pollution. However, for almost half (46 %) of the population, exposures and risks may be overestimated using a residence-based approach. Additionally, although the average error was found to be 3.6 %, the maximum (absolute) error was 64 %. Hence, exposure estimation methods that account for spatiotemporal changes in location and concentration may be needed for more accurate estimation of exposure and better health impact assessments. Nonetheless, this does not discount the importance of exposures at the residence location. Our results above, on the large percentage of time spent at the residence location (on average) and on the predictive value of residence urbanicity, are consistent with epidemiological studies that continue to suggest the value of exposures at residence location as a predictor for health responses (Brauer et al. 2008; Gan et al. 2011).

### Social distribution of exposure error

**Fig. 7:**
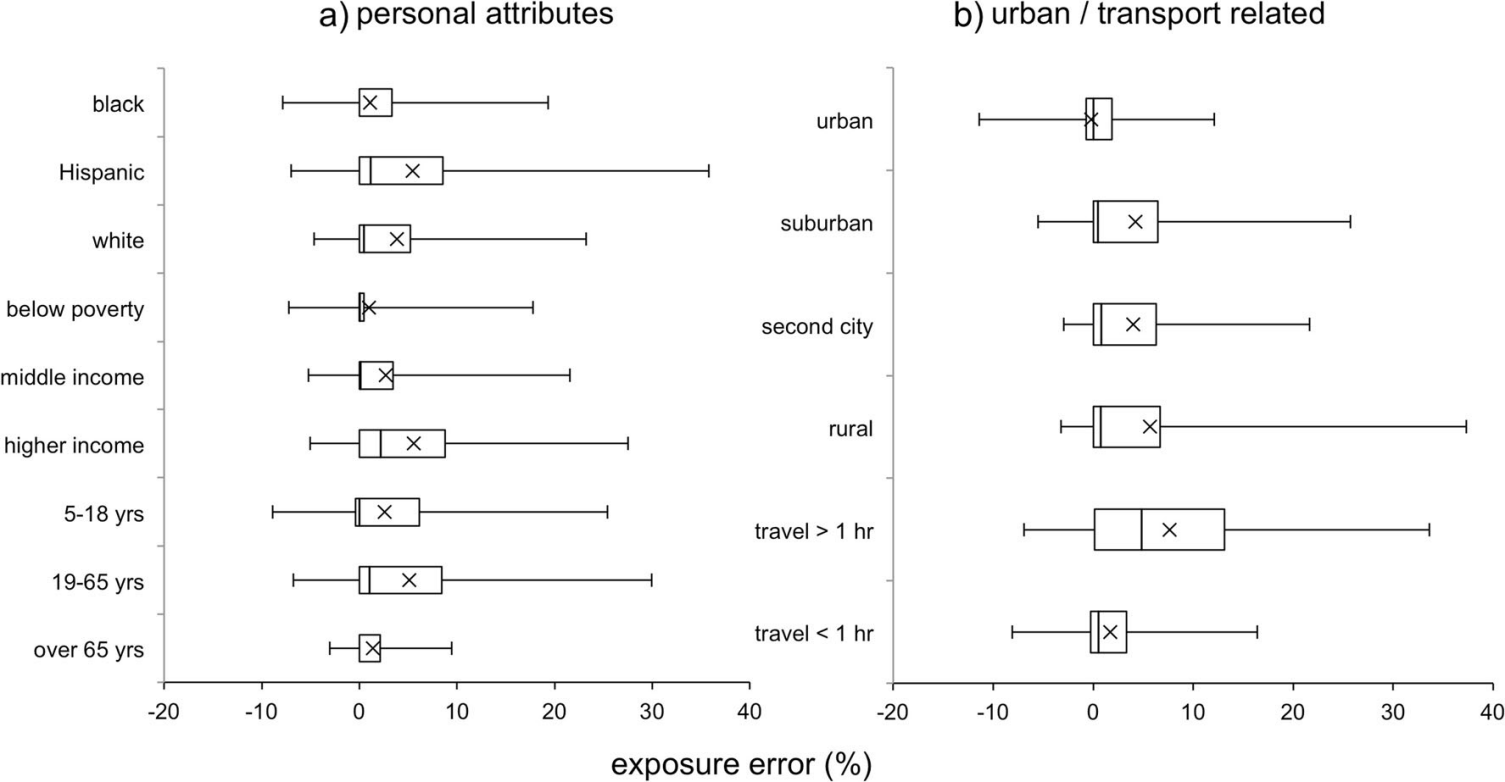
Cumulative distributions of exposure error for population subgroups related to **a** personal attributes and **b** urban characteristics. Middle income refers to households with income above the poverty threshold but with incomes less than $75 thousand (*k*). Note that the racioethnic subgroup populations are not exclusive, and populations have overlapping individuals

It is interesting to inquire whether estimated exposure error differs between demographic groups, i.e., whether residence-based estimates may be systematically biased for specific segments of the population; systematic biases could lead to systematic misclassification of exposures by group during health impact analyses. To address this question, Fig. 7 provides the cumulative distributions of exposure error for each of the sociodemographic groups studied above, with statistics provided in Table 2.

Among the racioethnic groups, exposure errors are largely positive (underestimation) for the Hispanic and white subpopulations, with the highest variation seen for the Hispanic group. Results are mixed for the black subgroup, with largely positive errors, but a substantial proportion in the negative (overestimation) range. Mean exposure errors were not found to be significantly different between any of the racioethnic groups considered. With regard to income, exposure errors are largely positive (underestimation) for the higher-income (annual income above $75,000) and middle-income groups, with some negative (overestimation) errors in the below poverty group. We found the mean exposure errors between the higher income group significantly different from those for both the below poverty group and the middle-income group, but difference between the low and middle income groups was not significant. Mean exposure error is positive for all age groups but is highest (most underestimation of exposures) for active adults (19 and 65 years) and lowest, with least variation, for the elderly (over 65 years). Mean errors were significantly different between these two age groups but not between either of these groups and the child (5–18 years) group.

Residential location appears to be a major determinant of the direction and the extent of exposure error. As can be observed from the box plots, the residence-based exposure concentrations are almost equal to the activity-based exposure concentrations for a large proportion (50 %) of those living in urban areas. This suggests that pollutant concentrations at residential and activity locations may be similar for those living in urban areas. Moreover, with decreasing density, the variability in the exposure error increases. Specifically, there is a greater incidence (and magnitude) of underestimation of exposures by residence-based estimates in rural regions (and overestimation in urban regions). Further, the mean exposure error was found to be significantly different for the individuals residing in urban regions and the individuals residing in the suburban, second city, and rural regions. These results suggest that using residence-based estimates may lead to underestimation of NO_x_ exposures (and resulting health effects) for those living in low-density regions, when compared to those in high-density urban areas.

Exposure error also increases, both in magnitude and variability, with an increase in the travel time. Further, an increase in the travel time leads to higher potential for underestimation of exposures. As such, ignoring activity-travel patterns for individuals who travel for a significant portion of their daily time could lead to the underestimation of health effect estimates.

In summary, the mean exposure errors are high for the age groups 19–65, above poverty groups, Hispanics, rural residents, and groups with travel time greater than 60 min. Specifically, the age-based differences in the exposure error may be a manifestation of the differences in the propensity to travel among the different age groups (children and elderly are likely to travel less). Within the context of income groups and rural residents, their travel patterns may be a contributing factor for the high exposure error (their daily activity patterns may lead them into more polluted areas compared to their residential locations). For the groups with longer travel times, spatial variation in concentrations could be a contributing factor for such large exposure error. These results suggest that residence-based estimates may underestimate exposures for the advantaged population groups, rather than vulnerable groups (with the exception of Hispanics).

Further, our results suggest that the residence-based approach may not necessarily lead to severely flawed exposure estimates for the most vulnerable subgroups of the population. This provides support for previous studies that did not consider activity-travel patterns in exposure analysis. In the absence of data on activity-travel patterns, such residence-based approaches may not necessarily lead to significantly biased exposure estimates, at least for a majority of the most vulnerable population segments. However, there are individuals within the susceptible groups who are still prone to either underestimation or overestimation of exposures using the residence-based approach. Additionally, the error may be important for people whose occupations require them to travel or be present for significant portions of time on roadways (e.g., sales personnel, highway workers, etc.).

To our knowledge, there is little previous literature on the socioeconomic distributions of exposure error within the USA. Limited evidence on this topic is available from Europe (Dhondt et al. 2012). While it is difficult to compare the social distributions of exposure error between these studies (as groupwise exposure errors are not reported in their study), we are able to observe a few similarities. Specifically, they also report that exposure error in rural locations is significantly higher compared to urban locations. Dhondt et al. (2012) also reported that rural zones had dynamic NO_2_ exposure values that could be 15 % higher than the static values. Our results above provide differences in the variability of exposure error between urban and rural regions and the distribution of exposure error among population subgroups.

### Limitations

Some limitations affect the robustness of these findings. First, the travel survey data used here may not be representative of the true spatiotemporal distribution of activities. Although the survey sample size is quite large, the county sample may not be large enough to capture the full spatial coverage necessary. Use of activity-based travel demand models for exposure analysis (Beckx et al. 2009; Dhondt et al. 2013; Dons et al. 2014; Hatzopoulou and Miller 2010) is one promising approach for generating the larger sample sizes that are needed. Second, exposures during travel activity were estimated using concentrations along the shortest route, as path data were not available. Although this is a reasonable approach, computed routes may not coincide with the actual travel paths on the particular sampled person-day. Third, due to limitations in the temporal availability of the input data sets, the travel data are from a 2009 survey, while the concentrations are based on the 2002 data. Hence, results are not expected to represent exposures for a particular year. Fourth, findings are limited by the use of estimated ambient pollutant concentrations for exposure analysis, rather than indoor, microenvironmental, or personal measurements. In the case of important indoor or personal sources, this could poorly represent exposures. Fifth, we have only directly considered one pollutant (NO_x_) in the analysis here. It is well known that spatial and temporal concentration patterns and scales of variability differ by pollutant (e.g., Bhugwant and Brémaud 2001). These differences could result in different distributions of exposure and exposure error. We expect the results here to be somewhat informative to understanding exposures to primary pollutants with substantial traffic emissions, but not to pollutants with substantial secondary formation or important emissions sources that are not collocated with traffic (such as ozone and formaldehyde). Sixth, defining urbanicity based on a single contextual population density measure could limit our findings. While this definition incorporates a few key characteristics of urban form, there is a need to consider additional measures including transportation infrastructure characteristics in defining urbanicity. Seventh, this work has focused on investigating inequality in exposures (and potential health outcomes) between population groups characterized by race, ethnicity, and income (and residence urbanicity). However, we note that there are many indices of social disadvantage and inequality that have been used in air pollution exposure and health impact studies; appropriate indicators likely depend on the social and political context. Further, there are many individual and group factors other than differences in exposures that can lead to differences in health outcomes (O’Neill et al. 2003); some of these are access to health care, overall health, smoking, diet, exercise, occupation, and genetics. Finally, it is well established that group averages do not necessarily represent the exposures of individuals in that group. Hence, the social disparity findings and implications can only address group level differences.

## Conclusions

In this study, we estimated ambient NO_x_ exposures for residents of Hillsborough County, FL, using activity-travel data (from the National Household Travel Survey) matched to the spatially resolved diurnal cycle of NO_x_ concentrations. Travel routes were estimated based on the shortest-time path. We examined the social distribution of these daily activity-based exposures. Finally, we compared our activity-based estimates with those that result from using only residence location.

### The findings of this work include the following:

- The Hillsborough County travel survey sample population spent little time in urban block groups. The time densities in urban block groups are larger for nonresidential than residential activities.
- The diurnal cycle of NO_x_ concentration in the study area exhibited typical morning and evening peaks, consistent with increased NO_x_ emissions from traffic during commute hours. Spatially, concentrations were highest near roadways and in urban areas throughout the day.
- The mean daily activity-based exposure concentration for the study sample was found to be 17 μg/m^3^, with values for individual person-day records ranging from 7.0 to 43 μg/m^3^.
- The black, Hispanic, and low-income subgroups had higher mean estimated activity-based exposures than comparison groups. The mean disparity in exposure between the black and white group is larger (4 μg/m^3^) than that between the below poverty and high-income group (2 μg/m^3^). However, regression results show that income below poverty is associated with a higher increase in exposure than black heritage alone, while Hispanic status was not found to be a significant predictor.
- The highest group mean exposure concentrations (22 μg/m^3^) were seen for those living in urban regions. Having an urban versus rural residence was also associated with the largest increase in exposure concentration in the regression (8.3 μg/m^3^). Furthermore, the residence urbanicity interaction variables largely explained the largest disparities found between sociodemographic groups. Being black while living in urban or suburban areas and living below poverty in suburban locations were each associated with higher exposures.
- Time in travel and other nonresidential activities was also associated with higher activity-based exposure concentrations, specifically 0.2 μg/m^3^ per hour spent away from home. This is due to the higher concentrations at these locations.
- The overall mean exposure error resulting from using residence-based versus activity-based estimation was 3.6 % here, with residence-based estimate lower for most of the sample population.
- The mean group exposure errors were highest for person-days with more than an hour of travel, people with higher household income, people living in rural areas, adults aged 19–65, and Hispanics. This suggests that studies that use residence-based exposure estimation may not be severely misclassifying exposures for disadvantaged and susceptible groups including blacks, low-income households, and elderly, at least on average.

In summary, this work demonstrates an approach for using available travel survey data and concentration modeling results for spatiotemporally resolved estimation of activity-based exposures. Novel contributions include the presentation and use of a spatially distributed activity time density map applied to exposure analysis and the examination of the social distribution of errors in exposure. Our results suggest that activity-based exposure estimation may be important for assessing exposures of individuals, but a residence-based approach may not necessarily lead to substantially biased exposure estimates for the most vulnerable groups, on average. Within the context of previous work, the results here continue to reveal the presence of social disparities in exposure, and possibly exposure-related health risks, in the study area, even after accounting for spatiotemporal population movement. Further, they confirm the importance of the urbanicity of residence location (and to a lesser degree, travel time) in influencing exposures and their social distribution. This supports the need for urban design policies that ensure that densification is accompanied by civil infrastructure (e.g., public transit) that decreases emissions in urban areas as well as time spent travelling, particularly for disadvantaged groups.
